# Clinical assessment of head injuries in motorcyclists involved in traffic accidents: A prospective, observational study

**DOI:** 10.1590/0100-6991e-20223340-en

**Published:** 2022-07-19

**Authors:** CRISTIANO BELOW, ISABELA CAMPOS BRIANTI, JOSÉ GUSTAVO PARREIRA, PEDRO DE SOUZA LUCARELLI-ANTUNES, NELSON SAADE, MURILO GOLIN, LUCA GIOVANNI ANTONIO PIVETTA, JOSÉ CARLOS ESTEVES VEIGA, JOSE CESAR ASSEF

**Affiliations:** 1 - Irmandade da Santa Casa de Misericórdia de São Paulo, Cirurgia - São Paulo - SP - Brasil; 2 - Faculdade de Ciências Médicas da Santa Casa de São Paulo, Cirurgia - São Paulo - SP - Brasil

**Keywords:** Craniocerebral Trauma, Accidents, Traffic, Tomography, Quality Assurance, Health Care, Traumatismos Craniocerebrais, Acidentes de Trânsito, Tomografia, Garantia da Qualidade dos Cuidados de Saúde

## Abstract

**Objective:**

to review the clinical assessment of head injuries in motorcyclists involved in traffic accidents.

**Method:**

prospective observational study, including adult motorcyclists involved in traffic accidents in a period of 12 months. Patients sustaining signs of intoxication were excluded. A modification of the Canadian Head CT Rules was used to indicate computed tomography (CT). Patients not undergoing CT were followed by phone calls for three months. Collected variables were compared between the group sustaining head injuries and the others. We used chi-square, Fisher, and Student’s t for statistical analysis, considering p<0.05 as significant.

**Results:**

we included 208 patients, 99.0% were wearing helmets. Seventeen sustained signs of intoxication and were excluded. Ninety (47.1%) underwent CT and 12 (6.3%) sustained head injuries. Head injuries were significantly associated with Glasgow Coma Scale<15 (52.3% vs. 2.8% - p<0,001) and a positive physical exam (17.1% vs. zero - p<0,05). Four (2.1%) patients with intracranial mass lesions needed surgical interventions. None helmet-wearing patients admitted with GCS=15 and normal physical examination sustained head injuries.

**Conclusion:**

Head CT is not necessary for helmet-wearing motorcyclists admitted with GCS=15 and normal physical examination.

## RESUMO

**Objetivo:**

análise crítica da investigação diagnóstica de lesões em segmento cefálico de motociclistas vítimas de acidentes de tráfego.

**Método:**

estudo observacional prospectivo incluindo motociclistas adultos vítimas de trauma, sem intoxicação exógena, em um período de 12 meses. A tomografia de crânio (TC) foi indicada de acordo com uma modificação dos “critérios canadenses”. Os pacientes que não foram submetidos a TC de crânio tiveram acompanhamento telefônico por três meses. A presença de lesões foi correlacionada com as varáveis coletadas através dos testes Qui-quadrado, t de Student ou Fisher, considerando p<0,05 como significativo.

**Resultados:**

dos 208 inicialmente incluídos, 206 (99,0%) estavam usando capacete. Dezessete estavam com sinais de intoxicação exógena e foram excluídos, restando 191 para análise. Noventa pacientes (47,1%) realizaram TC e 12 (6,3%) apresentaram lesões craniencefálicas, que se associaram significativamente a Escala de Coma de Glasgow (ECG) <15 (52,3% vs. 2,8% - p<0,001) e alterações ao exame físico da região cefálica/neurológico (17,1% vs. zero - p<0,05). Quatro pacientes (2,1%) precisaram tratamento cirúrgico de lesões intracranianas. Nenhum dos pacientes admitidos com ECG 15, em uso de capacete e sem alterações no exame físico apresentou TC alterada.

**Conclusões:**

para pacientes admitidos com ECG 15, que utilizavam o capacete no acidente e não apresentavam quaisquer alterações no exame físico, a realização da TC de crânio não trouxe mudanças no atendimento ao paciente. .

## INTRODUCTION

Trauma is an important cause of morbidity and mortality and loss of productive years of life in Brazil^1^. Many of these cases are of motorcyclists involved in traffic accidents^2^ ^,^ ^3^. There are several reasons that explain this fact, such as the country’s economic dynamics and vulnerability to the trauma mechanism^4^.

Traumatic brain injury (TBI) is the most frequent cause of trauma deaths. There are potentially lethal intracranial injuries that may go unnoticed on initial examination^2^. Thus, cranial computed tomography (CT) is liberally indicated to avoid delay in diagnosis^5^. However, since most trauma cases are mild, we observe head injuries in less than 15%^6^. There is a significant number of negative CTs, which results in lower method availability and higher cost, in addition to the excessive use of ionizing radiation^7^ ^-^ ^9^.

Objective criteria for requesting CT in trauma victims were developed, such as the NEXUS II, the New Orleans criteria, and the Canadian Head CT Rules^10^ ^-^ ^12^. Their application directs CTs to patients with a greater chance of injury. However, these studies were carried out in different scenarios than those observed in our country. The use of helmets, for example, is optional in some states of the USA, while mandatory in Brazil^13^. The use of such protocols in other realities is possible, but would need validation^14^.

The objective of this study is to perform a critical analysis of the diagnostic investigation of injuries in the cephalic segment in motorcyclists victims of traffic accidents.

## METHODS

This study was approved by the Ethics in Research Committee of our institution (CAAE 59539216.9.0000.5479). We carried out a prospective, observational study, from October 2017 to June 2018, including all motorcyclists over 18 years of age, victims of traffic accidents, admitted to the emergency room who agreed to the terms of the Informed Consent Form. (ICF). We excluded patients with clinical signs of exogenous intoxication.

To calculate the sample size, we used data from a previous study from our service^15^. In a period of 12 months, 1,346 trauma victims were treated at the emergency room, of whom 210 (15.6%) were motorcyclists. In nine months (period of the current study), 1,009 patients would be treated proportionally, 157 motorcyclists. For a tolerable sampling error of 5% and a confidence level of 95%, a sample considered reliable would have 112 cases or more.

We collected data on vital signs at admission, trauma mechanism, helmet use, CT scan performance and results, associated injuries, and treatment performed. Cranial CT was indicated for these patients by the highest-ranking physician, based on a modification of the Canadian Criteria^12^, according to the protocols of our service: patients with a Glasgow coma scale (GCS) less than 15 after two hours of trauma, clinical suspicion of skull fractures, signs of skull base fractures, more than one episode of vomiting, age over 65 years, amnesia greater than 30 minutes, “dangerous” trauma mechanism (vehicle ejection or associated run over), presence of seizures, and use of anticoagulants/platelet antiadhesives. We stratified the severity of intracranial injuries with the Abbreviated Injury Scale (AIS). We also analyzed its frequency, severity, and treatment.

Follow-up was performed for up to three months after trauma, via telephone contact. Patients or family members were asked about symptoms or complications related to TBI, especially about the need for a new hospital admission. Those who manifested suspicious symptoms would be called for medical reassessment.

We analyzed the data obtained from the research protocol using the SPSS 22.0 Software (IBM), comparing the quantitative data with the Student’s t test, and the categorical data, with the chi-square or Fisher’s test, when necessary, adopting as statistically significant the values of p<0.05.

## RESULTS

**Figure 1:**
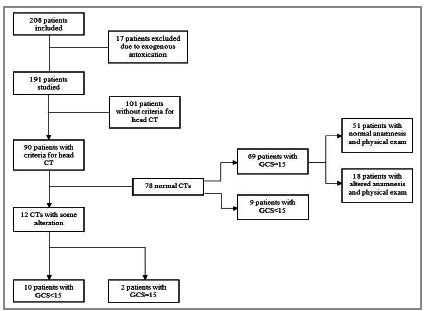
Flowchart of patients, performance of computed tomography, and its positivity.

A total of 208 patients met the inclusion criteria. Most motorcyclists were wearing helmets regularly (206 - 99.0%). The two patients who were not wearing a helmet presented with a lowered level of conscience at admission. There were 17 exclusions due to the presence of exogenous intoxication evident on physical examination (Figure 1).

The remaining 191 formed our sample, composed of 181 (94.0%) males, all wearing helmets (Table 1). GCS was 15 in 179 (93.7%) patients, of whom 24 (12.6%) had loss of consciousness, 18 (9.4%) mental confusion, four (2.0%) had amnesia, three (1.6%) vomiting, and one (0.5%) had dizziness. Indirect signs of skull base fracture were present in six patients (3.1%), and the cervical spine examination was altered in only one (0.5%).

**Table 1:** Data from the sample of 191 motorcyclists who were victims of traffic accidents.

Variable	n (%)
Male	181 (94.8%)
GCS=15	179 (93.7%)
Loss of consciousness	24 (12.6%)
Mental confusion	18 (9.4%)
Amnesia	4 (2.0%)
Dizziness	1 (0.5%)
Vomiting	3 (1.6%)
Skull Base Fracture	6 (3.1%)
Facial/Skull Hematoma	13 (6.8%)
Altered cervical exam	1 (0.5%)
Other fractures	77 (37.0%)
Cranial CT performed	90 (47.1%)
Positive cranial CT	12 (6.3%)
Surgical treatment	4 (2.0%)

GCS: Glasgow coma scale; CT: computed tomografy scan.

Computed tomography of the head was performed in 90 patients (47.1%) (Table 1). Of those, 19 had an GCS<15 and the remaining 71 underwent CT based on other criteria. Twelve (6.3%) had alterations on CT: facial fracture in six (3.1%), subdural hematoma in four (2.1%), skull fracture in three (1.6%), epidural hematoma in three (1.6%), subarachnoid hemorrhage in three (1.6%), cerebral contusion in two (1.0%), and cerebral edema in one (0.5%). Four (2.1%) patients required surgical treatment, three drainages of extradural hematoma and one drainage of subdural hematoma.

**Figure 2:**
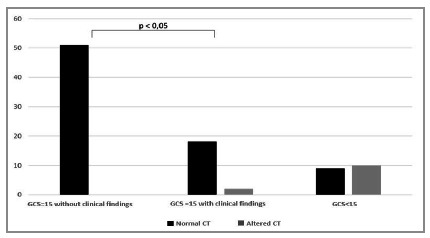
Comparison of computed tomography findings according to the Glasgow Coma Scale and physical examination findings in the cephalic segment.

Head injuries were significantly related to GCS<15 (52.3% vs. 2.8%, p<0.001). All patients without helmets had lesions in the cephalic segment (2/2), compared with 11.4% (10/88) of those with helmets (p=0.17) (Figure 2). In patients with altered physical examination in the cephalic region, CT identified lesions in 17.1% (12/70) of the cases, which did not occur in any of the patients without alterations in the physical examination (p=0.047). Of the motorcycle riders who wore helmets, arrived with a normal physical examination, and had GCS 15, none had head injuries (Figure 2).

There were no readmissions during the evaluated period. Of the 179 patients admitted with GCS=15 (including all who did not undergo a cranial CT), 160 (89.4%) were contacted by telephone, none of whom presented neurological complaints related to the trauma.

## DISCUSSION

Among motorcyclists involved in traffic accidents, 8.2% had clinical evidence of exogenous intoxication. If we consider that these patients are on public roads as drivers, this is a significant and worrying number. Of the 191 patients followed prospectively, most were male, with a mean age of 31.7 years, hemodynamically stable and without GCS changes at admission. About 99% wore a helmet, which admittedly reduces the frequency and severity of intracranial injuries^16^ ^-^ ^19^. Only 12 (6.3%) had lesions in the cephalic segment. However, four (⅓ of patients with lesions) required operative treatment. That is, although infrequent, these injuries cannot be underestimated.

The development of protocols for the identification of intracranial injuries in trauma victims is fundamental^20^ ^,^ ^21^. One of the biggest problems in trauma care is unnoticed injuries^2^. Regarding TBI, the situation of victims admitted with a normal level of consciousness and who progressively deteriorate to death if not treated properly (Talk-and-Die syndrome) is well known^2^. Due to CT availability, this complication is increasingly rare, but still present. On the other hand, the exaggerated use of CT may compromise diagnosis efficiency, resulting in many negative exams^6^. This is the reason for the use of objective criteria for requesting cranial CT in trauma patients.

The big three indication protocols for head CT, the Canadian CT Head Rules, the New Orleans Criteria, and the NEXUS II protocol, were developed in countries with significant differences in relation to Brazil^4^ ^,^ ^9^ ^,^ ^11^ ^,^ ^16^. Traffic legislation, the power and speed of motorcycles, as well as the roads and health system cannot be compared. The blind application of these protocols in our country can bring different results.

The Canadian criteria are the method described in the Advanced Trauma Life Support (ATLS 10^th^ edition) course for decision making in trauma victims with mild TBI (GCS 13-15)^22^. One of the biggest problems in adapting this rule to our environment is the interpretation of the “dangerous” mechanism (i.e. ejection of the vehicle). For motorcyclists who are victims of traffic accidents, ejection is frequent^4^. However, most cases in large cities occur on public roads, at low speeds, and with individuals protected by helmets. The systematic performance of CT ends up resulting in a significant number of negative exams, which results in an overload for the service and the health system. Therefore, we developed a modification of the Canadian Criteria in our service, based on previous studies^23^ ^,^ ^24^.

In our sample, we observed a significantly higher incidence of lesions in patients with a GCS less than 15 (52.3%), which confirms the performance of CT in this group. In patients with GCS=15, the divisor was the presence of changes in anamnesis and/or physical examination, identifying a subgroup with a higher chance of cranioencephalic injury. None of the patients with GCS=15 who wore a helmet and showed no changes in physical examination and/or anamnesis had any internal injuries. Remote follow-up was important to ensure that there were no misdiagnoses, even in the group that had not undergone head CT.

Thus, the model used in this study, which combines the assessment of clinical variables with physical and neurological examination in the trauma room, was able to identify important injuries in all cases. In many basic health units without a CT scanner, this model can identify cases to be transferred for complete diagnostic evaluation. It is important to note that, even with the selection criteria adopted, we still had 78 normal exams (40.8%). The opening remains for the evaluation of stricter selection criteria for CT, without compromising the sensitivity of the method.

The most significant limitation of our study is the sample size, which is still small considering the frequency of intracranial injuries. On the other hand, prospective horizontal monitoring, associated with strict inclusion criteria, aid in results credibility. Telephone follow-up was important to exclude the possibility of complications after hospital discharge.

Or results suggest that motorcyclists who were victims of traffic accidents wearing helmets, admitted with an GCS=15, without symptoms at the time of admission and with a normal physical examination, do not need a head CT scan in their initial evaluation, even if ejected. It is important to note that monitoring symptoms and warning signs for 48 hours is recommended for these cases. If there is any suspicion, CT should be performed.
